# Genotypic diversity and cariogenicity of *Candida albicans* from children with early childhood caries and caries-free children

**DOI:** 10.1186/s12903-015-0134-3

**Published:** 2015-11-17

**Authors:** Rongmin Qiu, Wenqing Li, Yan Lin, Dongsheng Yu, Wei Zhao

**Affiliations:** Department of Pediatric Dentistry, Guanghua School of Stomatology, Hospital of Stomatology, Guangdong Provincial Key Laboratory of Stomatology, Sun Yat-sen University, Guangzhou, China; Department of Pediatric Dentistry, College of Stomatology, Guangxi Medical University, Guangxi, China; Department of Dentistry, Jiangmen Central Hospital, Jiangmen, China; Department of Oral and Maxillofacial Surgery, Guanghua School of Stomatology, Hospital of Stomatology, Guangdong Provincial Key Laboratory of Stomatology, Sun Yat-sen University, Guangzhou, China; Guanghua School of Stomatology, Guangdong Provincial Key Laboratory of Stomatology, Sun Yat-sen University, 56 Ling Yuan Road West, Guangzhou, Guangdong Province 510055 China

**Keywords:** *Candida albicans*, Cariogenicity, Early childhood caries, Genotype

## Abstract

**Background:**

The genotypic diversity and cariogenicity of *C. albicans* from the dental plaque of children are poorly understood. This study aimed to explore the genotypic diversity and cariogenicity of *C. albicans* from children with early childhood caries and caries-free children.

**Methods:**

Dental plaque samples from 238 children with early childhood caries and from 125 caries-free children were collected for *C. albicans* isolation. A PCR method based on 25S rDNA was used to analyze *C. albicans* genotypes, and the strains with different genotypes were tested with regard to acidogenicity and aciduricity.

**Results:**

Among 129 *C. albicans* isolates, 79 (61.2 %) belonged to genotype A. The distribution frequency of genotypes A and C or genotypes B and C showed no significant difference between children with early childhood caries and caries-free children (*p* = 0.178 and 0.148), whereas genotypes A and B exhibited significantly different distributions (*p* = 0.010). No significant differences in aciduricity were found among the three genotypes, but the acidogenicity of genotypes B and C differed significantly from that of genotype A at pH 4.0.

**Conclusions:**

The genotypic distribution of *C. albicans* is associated with the caries experience of children, and the genotype may be related to its acidogenicity at pH 4.0.

## Background

Early childhood caries (ECC) is defined as the presence of 1 or more decayed teeth (noncavitated or cavitated lesions), missing teeth (due to caries), or filled tooth surfaces in any primary tooth in a child 71 months of age or younger [1]. Due to the high prevalence in primary school children, ECC is a public health problem of great concern. Because caries are caused by microorganisms in dental plaque, knowledge about the relationship between the microorganisms in children’s dental plaque and ECC is important for ECC prevention.

*C. albicans* is often detected in children with dental caries, while this yeast is typically absent in caries-free children [2, 3]. These findings provide indirect evidence for the association of *C. albicans* with dental caries. In our previous study, the frequency of *C. albicans* isolated in dental plaque from children with ECC was confirmed to be higher than that in caries-free (CF) children (44.1 vs. 19.2 %, *χ*^2^ = 22.213, *p <* 0.001), which indicated that *C. albicans* is related to ECC [4].

Some reports have analyzed the genotypic distribution of *C. albicans* in the dental biofilm of primary school children with different caries statuses and found that a specific genotype was dominant in the dental biofilm of children with severe early childhood caries (S-ECC) [5, 6]. However, the correlations between different *C. albicans* genotypes and the cariogenicity of this yeast remain unknown.

Researchers have found that the virulence factors of *C. albicans,* such as invasiveness and drug resistance, are linked to the genotype [7–9] and that acidogenicity and aciduricity are important *C. albicans* virulence factors for dental caries. In our previous study, the *C. albicans* strains isolated from the dental plaque of ECC children were found to be more aciduric than those from CF children, indicating that the aciduricity of clinical *C. albicans* strains is associated with a child’s dental status. In contrast, no differences were found with regard to acidogenicity between the two groups [10]. Regardless, the relationships of acidogenicity and aciduricity with genotype remain unclear.

In this study, we hypothesized that the genotypic distribution of *C. albicans* from ECC and CF children differs and that different *C. albicans* genotypes exhibit variable acidogenicity and aciduricity. To test this hypothesis, *C. albicans* from the dental plaque of ECC and CF children was detected by 25S rDNA-based PCR, and the acidogenicity and aciduricity of different *C. albicans* genotypes were evaluated.

## Methods

### Subjects

In total, 363 children aged 3–5 years (mean ± SD, 3.2 ± 1.9) were randomly recruited from four kindergartens in Guangzhou, China, from Feb. to Apr. 2009 (200 boys and 163 girls). The children were healthy and had no history of antibiotic use for at least 1 month before the study, and none of their permanent teeth had yet erupted. The children were assigned to one of two groups according to their caries status: the ECC group (*n* = 238) or the CF group (*n* = 125). The diagnostic criteria for ECC used in this study were proposed by Drury et al. [1]. The parents of children were fully informed in writing and written informed consent for participation was obtained from the parents who decided to take participation in the study.

Approval was obtained from the Research Ethics Committee of Sun Yat-sen University before the study began.

### Sample collection

Sample collection was performed in the morning, and the children were asked not to eat for 2 h before sample collection. Sterilized dental explorers were used for dental plaque collection. For the ECC children, dental plaque was obtained from caries lesions in the anterior teeth and/or molar teeth and then pooled. For the CF children, pooled dental plaque was obtained from the sound labial surfaces of upper anterior teeth and from the sound buccal surfaces of the upper and lower first primary molars. All samples were kept individually in sterile tubes containing brain heart infusion broth (HKM Co., Guangdong, China) and stored on ice; the samples were transported to the laboratory within 2 h after collection.

### *C. albicans* isolation and identification

All samples were dispersed by vortexing for 30 s to disperse any yeast aggregates. A 20-ml aliquot of each sample was inoculated onto a plate containing selective medium (CHROMagar Candida; CAC, CHROMagar Co., Paris, France) and then cultured for 48 h at 37 °C [11]. To obtain as many representative genotypes of *C. albicans* in one sample as practically possible, we selected 5 colony-forming units (CFUs) per sample (approximately 30–300 CFUs per child) from each CAC plate using a method described previously [12] to minimize selection bias and to maximize randomness. All the colonies in each sample were transferred to Sabouraud dextrose broth (SDB) (HKM Co., Guangdong, China) containing glycerol (30 % *v⁄v*) and preserved at −80 °C.

The methods of subject recruitment, sample collection, and *C. albicans* isolation and identification were described in our previously published study [4]. The isolates of *C. albicans* were the stored isolates from the previous study [4].

### DNA extraction

A boiling method was used for DNA extraction. Frozen suspensions of *C. albicans* strains were blended, and a 200-μl aliquot of each sample suspension was added to an Eppendorf tube and centrifuged for 8 min at 12,000 rpm. After the precipitate was ground in liquid nitrogen, it was allowed to stand for 5 min after the liquid nitrogen had evaporated; this procedure was repeated in triplicate. The precipitate was suspended in 30 μl of DNA extraction solution, boiled at 100 °C for 10 min, and centrifuged at 12,000 rpm for 3 min. The supernatant was stored at −20 °C until PCR amplification.

### PCR amplification

The primers CA-INT- L (5′-ATAAGGGAAGTCGGCAAAATAGATCCGTAA-3′) and CA-INT-R (5′-CCTTGGCTGTGGTTTCGCTAGATAGTAGAT-3′) were designed as previously described [13]. The amplification mixture (total volume, 50 μl) contained 10 μl of PCR buffer; 1 μl each of dNTPs, primers, and Taq polymerase (Promega, Madison, WI, USA); 34 μl of ddH_2_O; and 2 μl of the template DNA. The amplification conditions were as follows: initial denaturation at 93 °C for 5 min, 40 cycles of denaturation at 93 °C for 30 s, primer annealing at 55 °C for 45 s, and extension at 72 °C for 45 s, with a final extension at 72 °C for 7 min. For identification, the PCR products were loaded onto 2 % agarose gels (Bio-Rad, Hercules, CA, USA) at 100 V for 30 min and then stained with an ethidium bromide solution. The *C. albicans* sequences were classified into five genotypes according to the band pattern: genotype A (450 bp), genotype B (840 bp), genotype C (450 and 840 bp), genotype D (1080 bp), and genotype E (1400 bp) [14, 15].

### Suspension preparation

Twenty strains of each *C. albicans* genotype were examined for acid production and acid tolerance. Frozen stocks of the strains were inoculated in SDB and grown at 37 °C with shaking (150 rpm) under aerobic conditions. After the yeast cells were cultured for 17–24 h, they were harvested in the late exponential growth phase [16] and washed twice with phosphate-buffered saline (PBS, containing 0.05 mM Na_2_HPO_4_/KH_2_PO_4_, pH 6.8). The cells were suspended in PBS and adjusted to an optical density (OD) of 1.0 at 540 nm (equivalent to 1 × 10^8^ cells/ml) for acid production and acid tolerance tests. The OD value was detected using an ultraviolet spectrophotometer (752S, Lengguang Tech. Co., Shanghai, China).

### Acid production and acid tolerance assays

Sterile SDB (containing 100 mM glucose) was used for the acid production and acid tolerance assays; the pH of the medium was adjusted to different initial pH values of 7.0, 6.0, 5.5, 5.0, 4.5, and 4.0 (pH S-25, Shanghai Precision & Scientific Instrument Co., China) using sterile 2 mM HCl or 4 mM NaOH [17]. Briefly, 50-μl aliquots of suspensions of each of the different genotypes at an OD_540_ of 1.0 were added separately to 5 ml of SDB, cultured for 48 h at 37 °C and then centrifuged at 4500 rpm for 8 min at 4 °C. The pH of the final supernatant was detected using a standard pH meter to determine acid formation. The ∆pH (equal to the initial pH value minus the final pH value) was used to assess acid production.

To evaluate growth under acidic conditions, the precipitate was washed 3 times in PBS and then diluted with 2 ml of PBS. Then, the turbidity was measured at 540 nm (OD_540_) using a spectrophotometer.

These experiments were performed in triplicate. The mean ∆pH and OD_540_ were each calculated from three replicate samples. A greater ∆pH value indicated a greater ability to produce acid, and a greater OD_540_ value indicated better growth and higher aciduricity.

### Statistical analysis

Data analysis was performed using SPSS 16.0 (SPSS, Chicago, IL, USA). The relationship between the genotypic diversity of *C. albicans* and different caries experiences was analyzed using chi-square tests. To avoid type I errors when comparing multiple groups via chi-square tests, a critical *p-*value-required correction was applied, and the *p-*value threshold was set to 0.017 in three-way comparisons. Differences in ∆pH and OD_540_ values among the three genotypes of *C. albicans* were analyzed by ANOVA. When a significant difference was found among the three genotypes, the *LSD-t* method was used to analyze the difference between two groups, and the level of significance for the statistical tests was set at 0.05.

## Results

### *C. albicans* genotypic distribution

*C. albicans* strains were isolated from 129 subjects (35.5 % of 363 children): 105 children with ECC and 24 CF children. Each child from whom *C. albicans* was isolated presented only one genotype, with genotypes A, B, and C being the three detected genotypes. Genotypes D and E were not detected. Among the isolates, 79 (61.2 %) belonged to genotype A; 20 (15.5 %), to genotype B; and 30 (23.3 %), to genotype C. Genotype A was the major component in both groups of children (56.2 % in the ECC group, 83.3 % in the CF group); the frequencies of genotype B were 19.0 % and 0, and the frequencies of genotype C were 24.8 and 16.7 %, respectively.

**Fig. 1:**
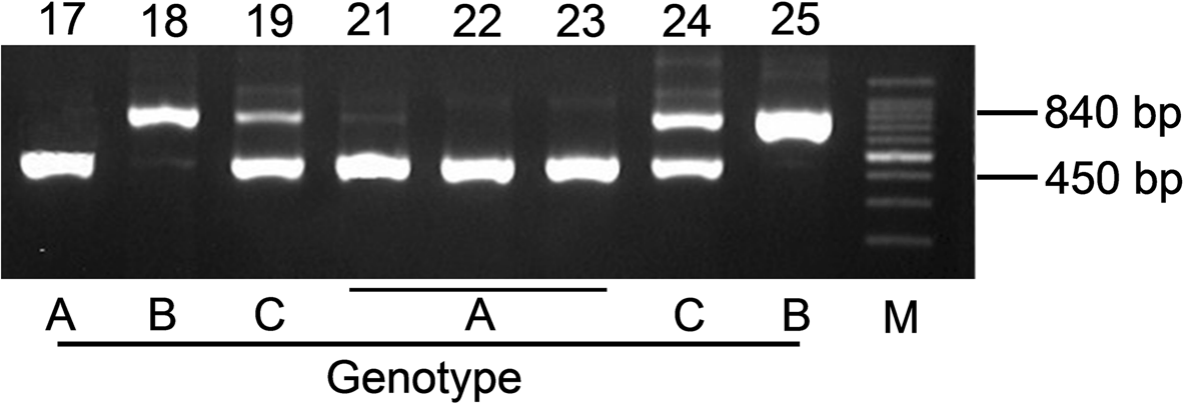
Different genotypic subgroups of *C. albicans* as determined by PCR. *Lanes 17*, *21*, *22*, and *23* show genotype A (the PCR amplification product is approximately 450 bp). *Lanes 18* and *25* show genotype B (the PCR amplification product is approximately 840 bp). *Lanes 19* and *24* show genotype C (two PCR amplification products: one band is approximately 450 bp, and the other band is approximately 840 bp)

The frequencies of different genotypic distributions in the ECC and CF groups were significantly different (*p* = 0.042). Genotypes A and B were distributed in a significantly different manner in the two groups (*p* = 0.010): genotype A presented a higher frequency in the CF group, and genotype B presented a higher frequency in the ECC group. The distribution frequencies of genotypes A and C or genotypes B and C showed no significant difference between the two groups, and the *p-*values were 0.178 and 0.148, respectively (Table 1 and Fig. 1).

**Table 1:** Genotypic distribution of *C. albicans* from dental plaque of children with different caries experiences, *n* (%)

Group	Total number of isolates	Genotype A	Genotype B^a^	Genotype C^b、c^	*p-*value
ECC	105	59 (56.2)	20 (19.0)	26 (24.8)	0.042
CF	24	20 (83.3)	0	4 (16.7)	
total	129	79 (61.2)	20 (15.5)	30 (23.3)	

^a^Comparison of the genotypic frequencies of A and B between ECC and CF groups (*p* = 0.010)

^b^Comparison of the genotypic frequencies of A and C between ECC and CF groups (*p* = 0.178)

^c^Comparison of the genotypic frequencies of B and C between ECC and CF groups (*p* = 0.148)

### Acidogenicity and aciduricity

The growth of *C. albicans* was gradually inhibited as the initial pH value of the medium decreased; however, *C. albicans* was able to grow at pH 4.0. As the initial pH of the cultures decreased, the OD_540_ values also decreased. The OD_540_ values for the *C. albicans* isolates of the three genotypes were not significantly different when cultured in media with different pH values (*p >* 0.05) (Table 2).

**Table 2:** OD values of different genotypes of *C. albicans* isolates (mean ± SD)

Genotype	OD_540_ value						
	4.0	4.5	5.0	5.5	6.0	6.5	7.0
A (*n* = 20)	1.41 ± 0.11	1.49 ± 0.10	1.52 ± 0.13	1.58 ± 0.14	1.58 ± 0.11	1.62 ± 0.14	1.63 ± 0.14
B (*n* = 20)	1.49 ± 0.13	1.53 ± 0.14	1.55 ± 0.14	1.57 ± 0.08	1.63 ± 0.10	1.65 ± 0.13	1.64 ± 0.16
C (*n* = 20)	1.49 ± 0.10	1.50 ± 0.07	1.54 ± 0.24	1.60 ± 0.15	1.61 ± 0.14	1.64 ± 0.14	1.64 ± 0.14
*p-*value	0.187	0.256	0.282	0.496	0.145	0.231	0.244

As the initial pH of the culture decreased, acid production by all *C. albicans* isolates was reduced. The final pH of all cultures ranged from 3.16 to 3.76. All strains exhibited the highest ∆pH values at pH 7.0. Among the three genotypes, no significant differences were found for ∆pH when cultured in media with pH values of 7.0, 6.5, 6.0, 5.5, 5.0, and 4.5 (*p >* 0.05). However, significant differences were found for ∆pH when the isolates were cultured in media at pH 4.0 (*p* = 0.029). After pairwise comparisons, no significant difference in ∆pH was observed between genotypes B and C (*p* = 0.836) when cultured in media at pH 4.0. In contrast, significant differences were found between genotypes A and B (*p* = 0.048) and between genotypes A and C (*p* = 0.019) (Table 3).

**Table 3:** Acid formation abilities of different genotypes of *C. albicans* isolates (mean ± SD)

Genotype	△pH value						
	4.0	4.5	5.0	5.5	6.0	6.5	7.0
A (*n* = 20)	0.84 ± 0.12	1.11 ± 0.11	1.46 ± 0.22	1.86 ± 0.19	2.37 ± 0.27	2.83 ± 0.20	3.27 ± 0.21
B (*n* = 20)	0.91 ± 0.09^a, b^	1.21 ± 0.20	1.55 ± 0.12	1.82 ± 0.39	2.49 ± 0.24	2.87 ± 0.40	3.39 ± 0.23
C (*n* = 20)	0.93 ± 0.03^c^	1.21 ± 0.08	1.64 ± 0.05	1.93 ± 0.26	2.57 ± 0.11	2.98 ± 0.15	3.38 ± 0.15
*p-*value	0.029	0.093	0.059	0.169	0.150	0.192	0.197

^a^Comparison of acid formation abilities of *C. albicans* isolates between genotype A and B cultured at pH 4.0 (*p =* 0.048)

^b^Comparison of acid formation abilities of *C. albicans* isolates between genotype B and C cultured at pH 4.0 (*p* = 0.836)

^c^Comparison of acid formation abilities of *C. albicans* isolates between genotype A and C cultured at pH 4.0 (*p* = 0.019)

## Discussion

*C. albicans* is detected more frequently in children with dental caries but is typically absent in caries-free children. This yeast can readily adhere to dental hard tissues in humans and form biofilms. This yeast also produces acid by metabolizing dietary sugars and carbohydrates, causing the dissolution of hydroxyapatite crystals in the enamel and dentin [16, 18]. *C. albicans* also produces acid at a pH value lower than 4.0 and shows strong acid tolerance [17].

To assess the genotypic identity of *C. albicans* strains, a PCR method based on 25S rDNA was employed for classification. To date, five genotypes of *C. albicans* have been identified, designated genotypes A, B, C, D, and E. Genotypes A, B, and C can be detected in the oral mucosa [15] and dental plaque [5, 6] of children. Genotype D is found in the periodontal pocket of systemically healthy patients with periodontitis [19], whereas genotype E is rarely present in the oral cavity. The results of this study revealed three genotypes of *C. albicans* strains in the examined children, genotypes A, B, and C, with the dominant strain being genotype A. These results are similar to a previous report showing that *C. albicans* genotype A is the dominant strain in the dental plaque of children [5, 6].

The genotypes of *C. albicans* from the oral cavities of different patients vary significantly possibly because the patients’ local oral conditions are unique [15]. In the present study, genotypes B and C were found more frequently in the ECC group than in the CF group. In particular, genotype B was not isolated from the CF group, whereas genotype A was more frequent in this group. Similar results were reported in previous studies, with genotypes B and C only detected in the carious site, not in the sound site, of S-ECC children [5] and with genotype B only detected in S-ECC children [6]. Such differences in the genotypic distribution of *C. albicans* may be related to differences in the sampling sites [5]. For the ECC children, the samples were obtained from caries lesions, whereas the samples for the CF children were obtained from sound surfaces. In the caries lesions, bad oral hygiene and high sugar concentrations in the cavities would have a greater influence on *C. albicans* colonization*.* Our findings suggested that different environments could facilitate the colonization of different genotypes of *C. albicans* and indicated that the distribution of *C. albicans* genotypes differed in children with different caries experiences.

In this study, the growth of *C. albicans* strains was gradually inhibited with a decreasing initial pH of the medium, and all *C. albicans* strains were able to grow at pH 4.0 and produce acid. However, the three genotypes did not significantly differ with regard to growth at any pH value, indicating that the three genotypes had similar acid tolerance and were all able to survive in a more acidic environment while continuing to produce acid. In this study, the acid-forming abilities of *C. albicans* genotypes B and C were significantly higher than the acid-forming ability of genotype A at pH 4.0 but not at pH 4.5–7.0. This result indicated that the genotype of *C. albicans* was related to its acidogenicity at pH 4.0. Notably, the pH value of the caries lesion is usually lower than that of the sound surface. The isolates of genotypes B and C were obtained from caries lesions, while some isolates of genotype A were obtained from sound surfaces. Therefore, the isolates of genotypes B and C would be able to survive in a more acid environment.

Moreover, *C. albicans* isolates of genotype A were detected more frequently in ECC children, and genotype A was less acidogenic at pH 4.0 than genotypes B and C. One reason for this finding is that *C. albicans* isolates were obtained from children with different caries status. *C. albicans* isolates from ECC children were found to be more acidogenic than those from CF children when cultured at pH 4.0 in our previous study [10]. In the present study, genotype A isolates were obtained from both ECC children and CF children, while all the genotype B isolates and most of the genotype C isolates were obtained from CF children.

Whether such genotypic diversity is related to the virulence of *C. albicans* remains uncertain. In patients with deep-seated infections, genotype A has been shown to be the most invasive isolate, whereas most non-invasive isolates belong to genotypes B and C [7]. Another study reported that of various clinical specimens, genotype C is the most invasive isolate, whereas most noninvasive isolates belong to genotype A [8]. However, in patients with bloodborne candidiasis, the genotypic distribution of *C. albicans* was not related to invasiveness [20]. In the present study, genotypes B and C were detected more frequently in the ECC group than in the CF group, and genotypes B and C showed significantly higher acid-forming abilities than genotype A when cultured at pH 4.0. In contrast*,* no relationship was found between the genotypic distribution of *C. albicans* and acid tolerance. Therefore, additional studies should be conducted to examine the relationship between cariogenicity and the genotype of *C. albicans*.

## Conclusions

In this study, genotypes B and C were found more frequently in the ECC group than in the CF group, indicating that the distribution of *C. albicans* genotypes differs among children with different caries experiences. The acid-forming abilities of *C. albicans* genotypes B and C were significantly higher than that of genotype A when cultured at pH 4.0, and this result indicated that the genotype of *C. albicans* is related to acidogenicity at pH 4.0.

## Abbreviations

*C. albicans*: *Candida albicans*
CF: Caries-free
CFUs: Colony-forming units
ECC: Early childhood caries
OD: Optical density
SDB: Sabouraud dextrose broth
S-ECC: Severe early childhood caries
